# Standardisation of Blood Pressure Measurement Among Patients Attending an Urban Primary Health Centre: A Quality Improvement Initiative

**DOI:** 10.7759/cureus.73811

**Published:** 2024-11-16

**Authors:** Baridalyne Nongkynrih, Ravneet Kaur, Thamizharuvi Muthukumarasamy, Nikhil Patil, Ankit Chandra, Ananda Selva Das

**Affiliations:** 1 Centre for Community Medicine, All India Institute of Medical Sciences, New Delhi, IND

**Keywords:** blood pressure measurement, quality improvement, standardised operating procedure, uhc, urban health centre

## Abstract

Accurate blood pressure (BP) measurement is essential for detecting, diagnosing, treating, and monitoring hypertensive individuals. This qualitative improvement initiative study is aimed at improving adherence to standardised BP measurement protocols in an urban primary health centre in Delhi, India. This study was conducted using a checklist based on World Health Organisation (WHO) guidelines. Baseline assessment revealed low adherence to standardised procedures. Interventions included crowd management, infrastructure and equipment, standard operating procedures (SOPs) development, and staff training. After two plan-do-study-act (PDSA) cycles conducted within six weeks, the adherence to standardised BP measurement significantly increased from 0.4% (n=1) to 86.2% (n=119). This study highlights the potential for targeted quality improvement in healthcare delivery.

## Introduction

Elevated blood pressure (BP) is a predominant risk factor for cardiovascular disease, affecting 1.28 billion adults aged 30-79 years globally [1]. Accurate measurement of blood pressure (BP) is essential for the detection, diagnosis, treatment, and ongoing monitoring of hypertensive individuals [2]. However, challenges such as technique-related errors and flawed clinic procedures, marked by substandard techniques and terminal digit preference, contribute to inaccuracies in readings [3-6]. The prevalence of hypertension among the Indian population is estimated to be 28.5% (n=3017) [7]. The accurate measurement of BP and the diagnosis of hypertension are crucial because misclassification can have serious clinical consequences [8]. Despite its widespread use, blood pressure measurement is susceptible to errors arising from inconsistent techniques, non-calibrated equipment, and inadequate staff training. Standardisation of measurement protocols becomes imperative to mitigate discrepancies and ensure precise cardiovascular assessments for all patients. Particularly in low- and middle-income countries, barriers like limited access to devices, power source challenges and insufficient training often lead to gaps in the appropriate measurement of blood pressure [9]. These hurdles are also discussed in a systematic review by Noa Kallioinen et al., which quantifies BP measurement inaccuracy under various headings: patient-related sources of inaccuracy, device-related sources of intimacy, procedure-related sources of inaccuracy and observer-related sources of inaccuracy [3].

Quality improvement (QI) is "a continuous and ongoing effort to achieve measurable improvements in the efficiency, effectiveness, performance, accountability, outcomes, and other indicators of quality in services or processes which achieve equity and improve the health of the community" [10]. QI initiatives are vital for recognising and rectifying gaps in the delivery of care [11,12]. QI has been used to increase the frequency of blood pressure measurement among neonates from 15.3%(n=167) to 54.7% (n=522) [13], increasing the adherence to standardised blood pressure measurement in primary health care settings to 71.6% (n=788) [14].

In our primary health centre, we found a gap in the standardised measurement of blood pressure. Through this QI initiative at the primary health centre, we aimed to increase adherence to the standardised procedure of blood pressure measurement from 0.4% to 70% within 6 weeks.

## Materials and methods

This initiative was conducted at the Urban Health Centre (UHC) Dakshinpuri Extension, in an urban resettlement colony in South Delhi. It was adopted as an urban field practice area by the Centre for Community Medicine, All India Institute of Medical Sciences, New Delhi. The UHC was set up as a primary-level healthcare facility to cater to the health needs of the community. The UHC caters to a population of 38,056, with 6000 households, as per the January 2023 census. It is spread over a total of 10 blocks. The number of patients visiting the UHC ranges from 400-600 per week. It provides outpatient services, BP, haemoglobin, fasting blood sugar, random blood sugar measurement, blood grouping, urine pregnancy tests and urine dipstick tests on all days except Tuesday and Sunday, along with antenatal care and immunisation services twice a week (Thursday and Saturday). These services are provided by a team of medical officers, laboratory technicians, pharmacists, health attendants, health educators and public health nurses. The blood pressure measurement was done by junior nurses who are in UHC for one month on rotation.

The treating physicians noted errors and inconsistencies in the blood pressure recordings of the patients, following which a team consisting of medical officers, treating physicians and public health nurses was formed. The baseline assessment was carried out for two weeks, from the last week of April to the first week of May 2023. Patients visiting the UHC during this period, who were above 18 years, including ante-natal women, were included in this assessment. For the baseline assessment, the QI team observed the procedure of blood pressure measurement of 255 patients using a checklist. All the participants were selected consecutively from the UHC for a period of four days of data collection. The checklist consisted of 11 items and was prepared by the team based on WHO Technical Specifications for Automated Non-Invasive Blood Pressure Measuring Devices with Cuff, a WHO medical device technical series released in 2020 [15]. A measurement fulfilling all the 11 criteria of the checklist was considered adherent to standardised procedures. For each observation, we allocated a score of one if it fulfilled the criteria, and zero if not. Therefore, each score ranges from 0 to 11.

**Figure 1:**
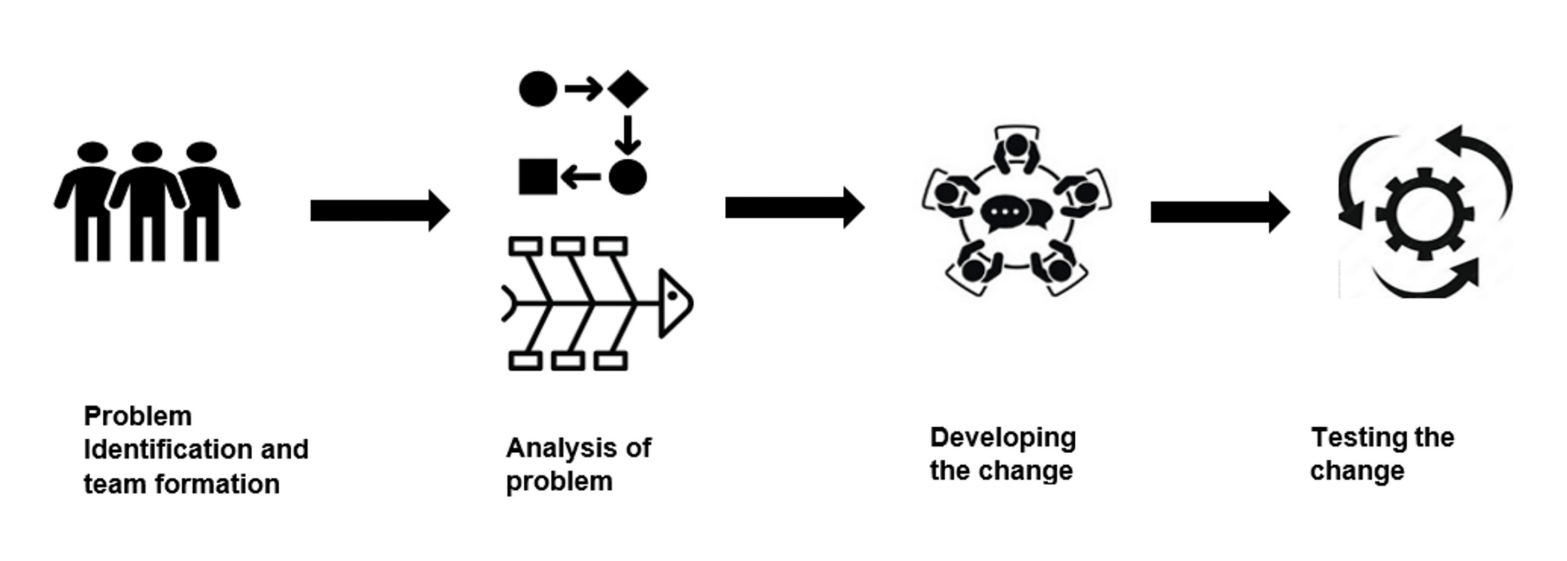
Diagram to explain the process of the study

To understand the problem in detail, we first mapped the existing process using a process flow diagram. Discussions were held with all members of the healthcare team involved in the management of patients with high blood pressure. A root cause analysis was done to identify the gaps in the existing process of blood pressure measurement, and the reasons for these gaps were ascertained (Figure 1).

Following this, brainstorming sessions were carried out and possible interventions (changes) were discussed. Thus, the change ideas were implemented in Plan-Do-Study-Act (PDSA) cycles. The checklist was filled in Google Forms using a smartphone. In the team, the medical officer had the role of leader and supervised the implementation of change, the treating physicians had the role of data reviewer and communicator to the staff of the health centre, while the public health nurse had the role of data collector and observed the BP measurement process.

Details of the changes

Crowd Management

Patients attending the UHC after registration were seated according to the availability of seats and directed to blood pressure measurement by the security guard. To avoid crowding near the blood pressure measurement area, the security guard was advised to let the patients in batches of 10 patients and seat them. Once the patients got their blood pressure measured and went in for consultation, the next set of registered patients were let in and seated in the queue for blood pressure measurement.

Sensitisation of the Guard as Crowd Manager

Regular discussions were held with the security guard to address crowd management challenges.

Infrastructure and Equipment

Stools were replaced with chairs to provide back support, and seating arrangements were optimised based on feedback from the UHC staff and faculty. An appropriate cuff for thin patients was purchased and made available at the screening counter.

Development of Standard Operating Protocols (SOPs)

Appropriate SOPs were developed.

Training of Health Workers

Nursing students on monthly rotations received brief training using a video created by the QI team, which was shown on their first day, and the SOPs were put up near the blood pressure measurement table. The training was done by the medical officer at the UHC. A public health nurse at the UHC performed data collection using the checklist. The treating physician of the team performed data analysis.

For the baseline, we did 245 observations, and for testing the change in the PDSA cycle, we selected around 120 observations of blood pressure measurement done consecutively at the UHC. A descriptive analysis was done using STATA 18 software (StataCorp. 2023. Stata 18. Statistical software. StataCorp LLC) and an unpaired student's t-test was used to assess the significant change in the outcome among the baseline and at the end of the PDSA cycle. Ethical approval was obtained from the Institute Ethics Committee of All India Institute of Medical Sciences (AIIMS), New Delhi, before starting the study (reference number: IEC-34/14.01.2022).

## Results

The QI team identified several gaps in the standardised measurement of blood pressure. As assessed on an 11-point checklist, it was found that less than 1% (n=2) of patients were given back support during measurements, and they were not queried about caffeine/nicotine intake or recent exercise prior to blood pressure assessment. Only 0.4% (n=1) of blood pressure measurements followed the standardised procedure (Table 1).

**Table 1:** The proportion of measurement having compliance with the standard procedure at the baseline The data has been represented as N (%). * Position the cuff on the patient’s bare upper arm (a light sleeve is acceptable) and centre it over the brachial artery (the centre is marked on most cuffs). The cuff should fit snugly on the arm, allowing no more than two fingers to fit between the distal part of the cuff and the skin. The distal part of the cuff should be positioned 1-2 cm above the cubital fossa.

S.No	Checklist	Baseline (n=245)
1	Asked the patient to abstain from caffeine, nicotine and exercise for 30 mins before BP measurement	2 (0.8%)
2	Asked the patient to relax ideally for 5 mins	45 (18.4%)
3	Patient sitting on a chair with their feet on the floor	141 (57.6%)
4	Patient sitting on a chair with their leg uncrossed	226 (92.2%)
5	Patient sitting on a chair with their back supported	1 (0.4%)
6	The patient was asked not to talk, read or use electronic devices during the rest period or during the assessment	199 (81.2%)
7	The observer was asked not to talk, read or use electronic devices during the rest period or during the assessment	182 (74.3%)
8	Appropriate size of cuff for patient’s mid-upper arm circumference	204 (83.3%)
9	Proper positioning of the cuff*	219 (89.2%)
10	Supported the patient’s arm so that the middle of the cuff is at heart level	244 (99.6%)
11	Recorded exact reading, didn’t round off	244 (99.6%)

**Figure 2:**
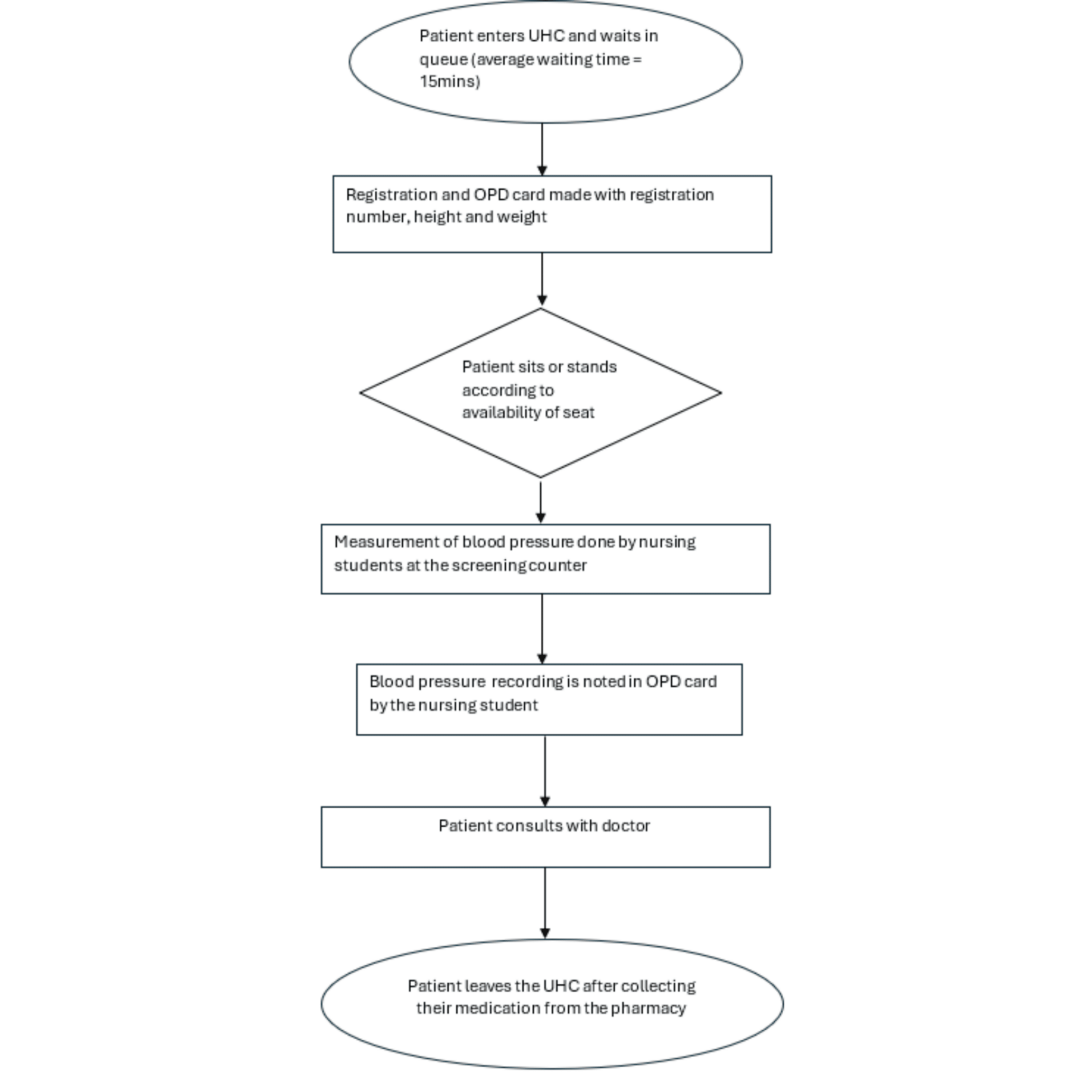
Process flowchart showing the patient pathway at the urban health centre

The mean baseline score for adherence to the standardised procedure of blood pressure measurement among 255 patients was 6.9 (SD-1.2), none (0%) of the observation had fulfilled all 11 checkpoints. The process flow chart was drawn to understand the micro-process, from registration to consultation to the persons visiting the UHC (Figure 2).

**Figure 3:**
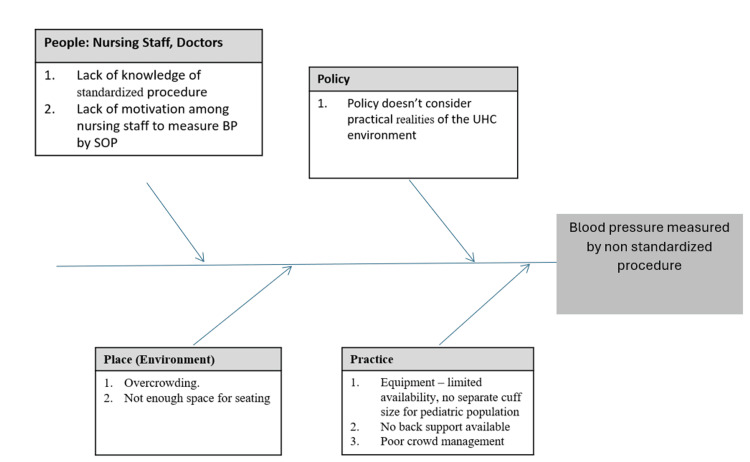
Root cause analysis

This highlighted the concern that patients are not given sufficient rest before their blood pressure is measured. Afterwards, a discussion was held with the UHC staff and nursing students, and a fishbone diagram was prepared to comprehend the root cause (Figure 3).

Through interviews with the patients and nursing students, various factors were identified such as patients' lack of awareness regarding the significance of BP measurement and their urgency to be seen by doctors and insufficient knowledge and motivation among nursing students to adhere to SOPs due to time constraints in a busy outpatient department (OPD) with limited equipment availability. Additional challenges included the unavailability of appropriate cuff sizes for paediatric and thin patients, as well as the absence of back support during BP measurements.

**Figure 4:**
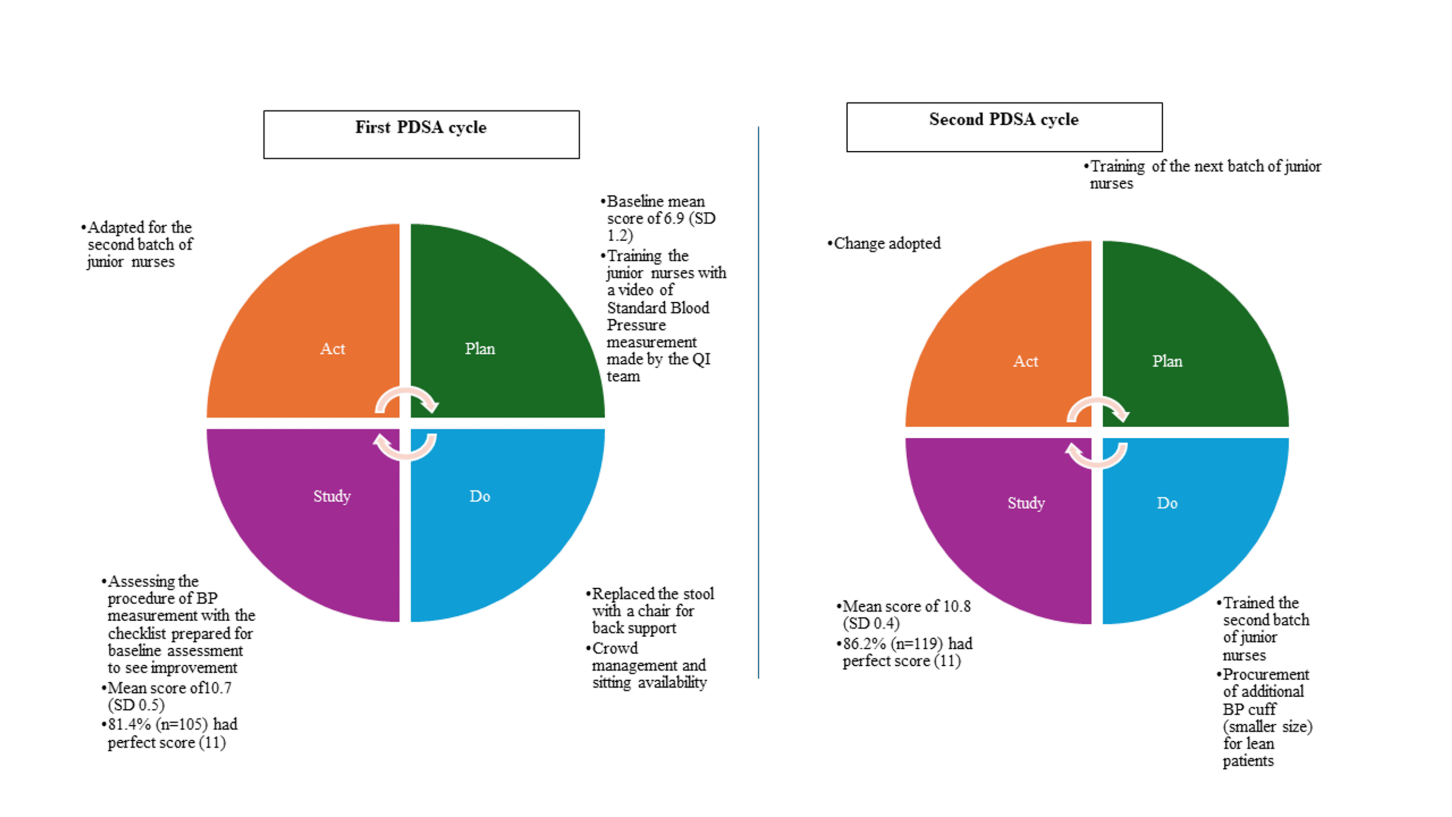
Plan-Do-Study-Act (PDSA) cycles carried out to test the change for increasing the availability of the patient health record among patients with non-communicable diseases in the urban health centre, Dakshinpuri, New Delhi

The above changes were implemented through the PDSA cycle (Figure 4).

**Figure 5:**
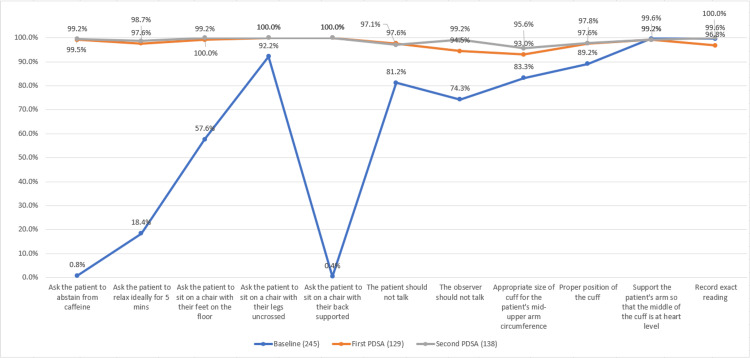
Improvement in the proportion of patients getting their blood pressure measured according to the standardised procedure in various components of the assessment checklist The data has been represented as %.

In the first PDSA cycle, we had 129 observations and the mean score for adherence to the standardised procedure for blood pressure measurement was 10.7 (SD-0.5). The change was statistically significant (p-value <0.001). A total of 81.4% (n=105) observations had complete adherence to the standardised procedure (fulfilled all the 11 points of the checklist). We ran a second PDSA cycle for the next batch of junior nurses in which an additional BP cuff for thin patients was also procured. The mean score for adherence to the standardised procedure for blood pressure measurement among 138 observations in the second PDSA cycle was 10.8 (SD-0.4), p-value >0.05 compared to the first PDSA cycle. A total of 86.2% (n=119) observations had complete adherence to the standardised procedure in the second PDSA. The details of the adherence to each component of the checklist are shown in Figure 5.

## Discussion

In this QI, we were able to increase adherence to the standardised procedure for blood pressure measurement from the mean score of 6.9 to 10.8, and the proportion of complete adherence from 0.4% (n=1)to 86.2% (n=119) within six weeks. We focused on strengthening primary health care services for patients visiting our urban primary health centre in Delhi by finding the gap, discussing the change ideas, running charts and following up by implementing change through a PDSA cycle [16]. The adherence to the standardised blood pressure measurement was lower in our setting as compared to the studies conducted by Shaikh et al. (Northern California in 2016-2018) [17], Flynn RS et al. (Philadelphia in 2019-2021) [13], and Zhang et al. (China in 2021-2022) [18]. Significantly, our study observed a more marked increase in adherence following the initial PDSA cycle. This enhancement can be attributed to the implementation of a specialised checklist and the training of young nursing students. Notably, younger students demonstrate higher receptivity to training and exhibit greater enthusiasm compared to more seasoned staff members [19]. However, this could be also due to the influence of some external factors like motivated staff and interest in the superiors. During this period, there were no changes in patient load or the work timings of the centre. In this study, we faced challenges due to time constraints and the frequent turnover of nursing students and security guards, necessitating ongoing training efforts for the successful implementation of our interventions. Time constraints within the busy OPD posed challenges to adherence to standard operating procedures by nursing students, highlighting the need for efficient protocols that accommodate time-sensitive patient care. Patients' lack of awareness regarding the importance of blood pressure measurement, coupled with their urgency to see doctors, contributed to difficulties in ensuring adherence to standardised procedures, emphasising the need for patient education initiatives.

The study's focus on improving adherence to protocols in a clinical setting underscores its practical relevance and potential impact on enhancing patient outcomes and safety. Integrating training for young nursing students highlights a proactive approach to addressing adherence issues, potentially leading to sustainable improvements in healthcare delivery. Continuous training and education sessions should be provided to healthcare personnel, particularly nursing students, to ensure adherence to standardised protocols. Emphasising the importance of following SOPs and providing regular updates on best practices can enhance staff competency and confidence.

One strength of this initiative is that both the baseline and post-intervention assessments were conducted using a standardised checklist based on WHO specifications [15]. This approach enhances the reproducibility and standardisation of interventions, thereby contributing to the reliability of the results. Another strength lies in our enthusiastic team, which included young and motivated members who were instrumental in implementing the changes. Employing a multidisciplinary team-based approach, we incorporated suggestions and feedback from various staff members to identify and address underlying issues. Through a series of PDSA cycles, the study implemented targeted interventions, including optimised seating arrangements, staff training and availability of appropriate equipment, resulting in a substantial increase in adherence to standardised procedures.

However, our work also has some limitations. Data collection relied on observational assessments conducted by a public health nurse, introducing the possibility of observer bias and subjectivity in recording adherence to standardised procedures. We were unable to assess the impact of adherence to the standardised protocol for blood pressure measurement on changes in blood pressure control status and the proportion of newly diagnosed patients with hypertension. Additionally, the duration of this study was relatively short, and the sustainability of the changes needs to be assessed in future studies. The study's findings may have limited generalisability due to its single-site nature, potentially affecting the applicability of the interventions to other healthcare settings.

## Conclusions

This initiative represents a unique effort aimed at improving the quality of blood pressure measurement standardisation in a primary healthcare facility in India. We increased the adherence to standardised procedures from the mean score of 6.9 to 10.8, and the proportion of complete adherence from 0.4% (1) to 86.2% (119) within six weeks. This highlights the potential for targeted quality improvement in healthcare delivery. However, further studies across diverse healthcare settings are needed to ensure sustainability in the future.
